# Endothelial Dysfunction in Patients Undergoing Cardiac Surgery: A Narrative Review and Clinical Implications

**DOI:** 10.3390/jcdd10050213

**Published:** 2023-05-13

**Authors:** Danijel Knežević, Božena Ćurko-Cofek, Tanja Batinac, Gordana Laškarin, Marijana Rakić, Maja Šoštarič, Marko Zdravković, Alan Šustić, Vlatka Sotošek, Lara Batičić

**Affiliations:** 1 Department of Anesthesiology, Reanimatology, Emergency and Intensive Care Medicine, University of Rijeka, Braće Branchetta 20, 51000 Rijeka, Croatia; danijel.knezevic2@uniri.hr (D.K.); alan.sustic@uniri.hr (A.Š.); 2 Department of Physiology, Immunology and Pathophysiology, Faculty of Medicine, University of Rijeka, Braće Branchetta 20, 51000 Rijeka, Croatia; bozena.curko.cofek@uniri.hr (B.Ć.-C.); gordana.laskarin@uniri.hr (G.L.); 3 Department of Clinical Medical Sciences I, Faculty of Health Studies, University of Rijeka, Viktora Cara Emina 2, 51000 Rijeka, Croatia; tanjabatinac@net.hr; 4 Hospital for Medical Rehabilitation of Hearth and Lung Diseases and Rheumatism “Thalassotherapia-Opatija”, M. Tita 188, 51410 Opatija, Croatia; rakicmarijana@yahoo.com; 5 Clinical Department of Anesthesiology and Perioperative Intensive Therapy, Division of Cardiac Anesthesiology and Intensive Therapy, University Clinical Center Ljubljana, Zaloska 7, 1000 Ljubljana, Slovenia; maja.sostaric@kclj.si; 6 Department of Anesthesiology and Reanimatology, Faculty of Medicine, University of Ljubljana, Vrazov Trg 2, 1000 Ljubljana, Slovenia; 7 Department of Anaesthesiology, Intensive Care and Pain Management, University Medical Centre Maribor, Ljubljanska ulica 5, 2000 Maribor, Slovenia; markozdravkovic@gmail.com; 8 Department of Medical Chemistry, Biochemistry and Clinical Chemistry, Faculty of Medicine, University of Rijeka, Braće Branchetta 20, 51000 Rijeka, Croatia; lara.baticic@uniri.hr

**Keywords:** anesthesia, cardiac surgery, endothelium, endothelial dysfunction, endothelial glycocalyx, intensive care

## Abstract

Cardiac surgery is one of the highest-risk procedures, usually involving cardiopulmonary bypass and commonly inducing endothelial injury that contributes to the development of perioperative and postoperative organ dysfunction. Substantial scientific efforts are being made to unravel the complex interaction of biomolecules involved in endothelial dysfunction to find new therapeutic targets and biomarkers and to develop therapeutic strategies to protect and restore the endothelium. This review highlights the current state-of-the-art knowledge on the structure and function of the endothelial glycocalyx and mechanisms of endothelial glycocalyx shedding in cardiac surgery. Particular emphasis is placed on potential strategies to protect and restore the endothelial glycocalyx in cardiac surgery. In addition, we have summarized and elaborated the latest evidence on conventional and potential biomarkers of endothelial dysfunction to provide a comprehensive synthesis of crucial mechanisms of endothelial dysfunction in patients undergoing cardiac surgery, and to highlight their clinical implications.

## 1. Introduction

Cardiac surgery involves procedures on the heart and thoracic aorta. It plays an important role in the treatment of heart diseases whose prevalence is continuously increasing [1]. Currently, more than a million cardiac surgeries are performed annually worldwide [2]. The indications for cardiac surgery are described in detail in the 2019 guidelines jointly produced by three associations: the European Association for Cardio-Thoracic Surgery (EACTS), the European Association of Cardiothoracic Anesthesiology and Intensive Care (EACTAIC), and the Quality and Outcomes Committee of the European Board of Cardiovascular Perfusion (EBCP) [3]. Overall, the most common heart pathologies that need surgical treatment are severe valvular stenosis or regurgitation and an advanced form of ischemic heart disease.

In patients with valvular heart disease, depending on the valve affected, surgical treatment includes valve reconstructions or replacement during open heart surgery [4]. In patients with an advanced form of the ischemic heart disease, when medical and/or invasive cardiological therapy are insufficient, cardiac surgery should be performed [5,6]. Possible treatment modalities include minimally invasive or open cardiac surgery [7,8].

Most cardiac surgeries are performed with cardiopulmonary bypass (CPB), which temporarily replaces the heart and lung functions with an artificial circuit consisting of a pump and an oxygenation membrane [1]. CPB allows a bloodless surgical field and quiescent heart while maintaining systemic perfusion and adequate oxygenation. Roller and centrifugal pumps on the CPB machine produce non-pulsatile flow, which is still the most frequent type of CPB [9]. Recently, the pulsatile flow has been introduced; it is thought to be more physiological because it mimics arterial pulsations. Nowadays, considerable efforts are being made to identify underlying mechanisms involved in organ dysfunction following cardiac surgery, and the non-pulsatile flow is considered one of them. Although the 2019 EACTS/EACTAIC/EBCP guidelines recommend the use of pulsatile flow during CPB in adult open-heart surgery, there is still a lack of evidence for its beneficial effect over non-pulsatile flow [3].

There are also some other mechanisms related to perioperative organ dysfunction in cardiac surgery, including the release of numerous mediators and vascular endothelial dysfunction. Inflammatory mediators such as interleukin (IL)-1, IL-6, IL-8, IL-12, and IL-18 are released due to the chronic inflammation of the myocardium caused by stenosis of the vessels and the surgical stress itself [10,11,12]. There is also a noticeable secretion of degradation products of the endothelial glycocalyx due to the activation of the pro-inflammatory cascade and the need for abundant volume compensation with the aim of maintaining hemodynamic stability during and after the procedure, which leads to the secretion of the atrial natriuretic peptide and consequently damage to the endothelial glycocalyx [13]. The moderation of resultant endothelial dysfunction has become a focus of clinical and animal research.

The disorders of endothelial glycocalyx are also detected in non-cardiac surgery, as anesthetics, fluid overload and ishemic-reperfuison injury can affect the degradation of endothelial glycocalyx. However, disorders of endothelial glycocalyx are more pro-nounced in the cardiac surgery where among others, the extensive contact of blood and the artificial circuits during the CPB lead to a prominent surgical stress response. Moreover, the patients undergoing cardiac surgery have higher endothelial dysfunction before the surgical procedure due to the immanent characteristic of their basic disease.

Thus, in this narrative review, we aim to present the mechanisms involved in vascular endothelial dysfunction and its clinical implications in patients undergoing cardiac surgery. We will also emphasize potential strategies for the protection and preservation of the endothelial glycocalyx during surgery, which could lead to improved patient outcomes.

## 2. Basic Structure and Function of Endothelial Glycocalyx

**Figure 1:**
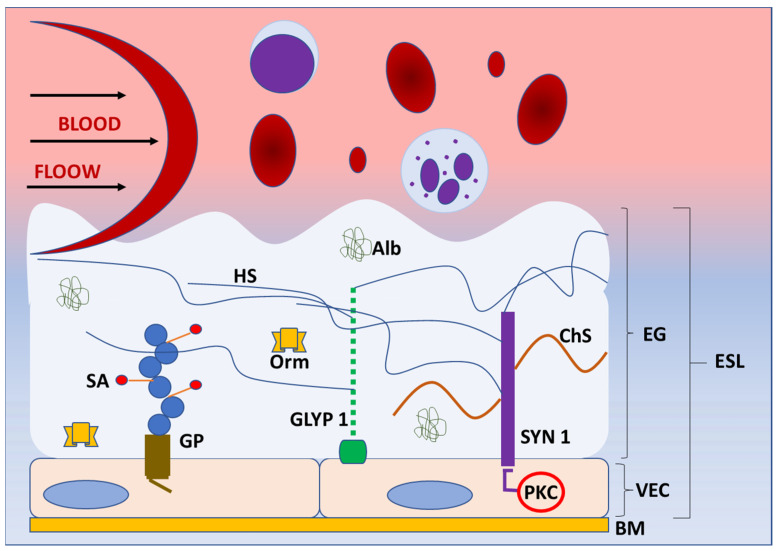
Structure of endothelial glycoaylyx. Schematic representation of the basic structure of endothelial glycocalyx (EG) under normal physiologycal conditions. EG forms a protective layer of glycosaminoglycans, proteoglycans (syndecans, glypicans) and incorporated proteins on the luminal side of vascular endothelial cells, preventing direct contact of blood elements with the blood vessel wall. The components of EG transmit intraluminal events to endothelial cells activating the enzymes (protein kinase C) and intracellular signaling pathways. Abbreviations: Alb—albumin, BM—basement membrane, ChS—chondroitin sulphate, EG—endothelial glycocalyx, ESL—endothelial surphace layer, GLYP 1—glypican 1, GP—glycoprotein, HS—heparan sulphate, Orm—orosomucoid, PKC—protein kinase C, SA—sialic acid, SYN 1—syndecan 1, VEC—vascular endothelial cell.

The blood vessel wall has three layers: tunica interna or intima, tunica media, and tunica externa or adventitia. The tunica interna is located next to the lumen and covered with one layer of endothelial cells attached to the basement membrane. These cells are in direct contact with blood components and form a barrier to the tissue. As such, endothelial cells exert numerous functions, including control of extravasation of fluids, ions, and molecules and regulation of vascular tone, blood coagulation, and leukocyte activation in the inflammatory and immune response [14]. Endothelial cells also produce components of the glycocalyx, which covers their luminal (apical) side [15]. The glycocalyx and attached plasma proteins, such as albumin, orosomucoid, antithrombin III and growth factors, form the endothelial surface layer (ESL) [16,17] (Figure 1).

Before the use of electron and confocal microscopy, the existence of the endothelial glycocalyx was unknown. Around 70 years ago, a thin structure was discovered that is known today as the endothelial glycocalyx, which prevents the direct contact of blood elements with the blood vessel wall [16,17]. Dr Stanley Bennett was the first who proposed the term endothelial glycocalyx for this extracellular polysaccharide-rich structure [18]. Glycoproteins and proteoglycans are the main components and the basic structure of the endothelial glycocalyx. Glycoproteins are glycosylated molecular complexes containing carbohydrate groups covalently attached to the protein by covalent bonds, whereas proteoglycans are proteins attached to at least one glycosaminoglycan chain. They both anchor glycocalyx to the vascular endothelial cells, creating a matrix with incorporated soluble and insoluble components [19]. Some of these components are plasma proteins, enzymes, cofactors, superoxide dismutase, antithrombin III, thrombomodulin, and xanthine-oxidoreductase [20]. Glycoproteins and proteoglycans are mostly cell adhesion molecules that consist of variable extracellular domains, a transmembrane domain, and a cytoplasmic tail and belong to selectin, immunoglobulin, or integrin receptor families [15]. It was also noticed that the endothelial glycocalyx acts as a filter for plasma proteins depending on their size and charge [18]. The glycoproteins have short carbohydrate side chains, which are capped with sialic acid [21]. The studies showed that sialic acid in the endothelial glycocalyx significantly contributes to its negative charge and that reduction of sialic acid content results in the reduction of vascular endothelium negative surface charge [21].

Proteoglycans bind long, negatively charged, hydrophilic, unbranched glycosaminoglycan chains of disaccharide units [22,23]. Some of the glycosaminoglycans are chondroitin sulphate (associated with syndecans), heparan sulphate (associated with syndecans and glypicans), hyaluronic acid (hyaluronan; binds to surface receptors, e.g., CD44), and dermatan sulphate (covalently attached to serine residues of core proteins) [24]. Heparan sulphates are the most abundant and comprise 50–90% of all glycosaminoglycans [25]. The sulfonation of glycosaminoglycans significantly contributes to the negative charge of endothelial glycocalyx, which allows the binding of proteins from blood [26]. Syndecans and glypicans are the most significant proteoglycans, along with biglycans, decorins, mimecans, and perlecans, which are all present in the endothelial glycocalyx. So far, there are four known syndecans—syndecan-1, -2, -3, and -4 [27]. The syndecans are incorporated into the cell membrane. Their cytoplasmic tails are in contact with protein kinase C and may initiate different intracellular signaling events [27]. Through the connections with proteins of the cytoskeleton, syndecans allow for the transmission of extracellular mechanical forces to the cell [28]. Additionally, they participate in the regulation of the inflammatory response in infection and trauma. Syndecans express many glycosaminoglycan chains, which bind cytokines and initiate the inflammatory response. Animal models showed the involvement of syndecans in various aspects of inflammation, from leukocyte recruitment to the resolution of inflammation. Furthermore, the upregulation of syndecan expression during inflammation and a direct relationship between serum syndecan level and severity of inflammation were reported in humans [27]. Although the role of syndecan-1 in inflammation is the most studied, other syndecans are also involved in the inflammatory response [29].

The glypican family has six members—glypican-1 to glypican-6. Unlike syndecans, which are transmembrane structures, glypicans are connected to the cell membrane via glycosylphosphatidylinositol molecules in the areas of lipid rafts rich in signaling molecules [30]. Glypican-1 consists of the core protein and three heparan sulfate chains. It is a coreceptor in many signaling pathways, such as vascular endothelial growth factor-A, transforming growth factor-β, and bone morphogenic protein. Hence, glypican-1 modulates those pathways through the interactions with ligands and receptors on the cell surface [31]. It is also involved in signaling pathways that result in the activation of endothelial nitric oxide synthase (eNOS) and nitric oxide (NO) production [32]. It was demonstrated that glypican-1 is overexpressed in various types of cancers (breast, pancreatic, glioma) and that its high level of expression correlates with poor prognosis [30]. Additionally, the glypican-1 isoform, as the component of the glycocalyx, has a significant role in shear stress mechanosensation and mechanotransduction. [31]. The endothelial glycocalyx protrudes in the lumen of the blood vessel, and it is constantly under the shear stress generated by blood flow. However, the endothelial glycocalyx also connects to the cell membrane and cytoskeleton and includes the molecules that activate signaling pathways, such as syndecans [27]. Hence, the endothelial glycocalyx translates blood shear forces to functional and genetic changes inside the endothelial cells [24]. The results of shear force sensing and transducing are eNOS activation, NO production, and vasodilatation [33]. The cell culture and animal model study by Mahmoud et al. showed that the inhibition of glypican-1 results in endothelial cell dysfunction and inflammation through enhanced inflammatory gene expression, monocyte adhesion, and inhibited NO expression [24].

Various pathogens can be present in the cardiovascular system and blood. Therefore, the endothelial glycocalyx is also exposed to these pathogens and protects endothelial cells by providing the physical distance barrier and preventing adhesion [15]. When bacteria enter the blood, they must penetrate the endothelial cells to colonize the tissue. Since most gram-negative and gram-positive bacteria have negatively charged surfaces, and the endothelial glycocalyx is also negatively charged, it repels pathogens and prevents their access to endothelial cells [34]. Regarding the viruses, they mostly have a negative surface at pH 7.4. Accordingly, the endothelial glycocalyx represents the electrostatic charge barrier for the viruses as well [35].

**Figure 2:**
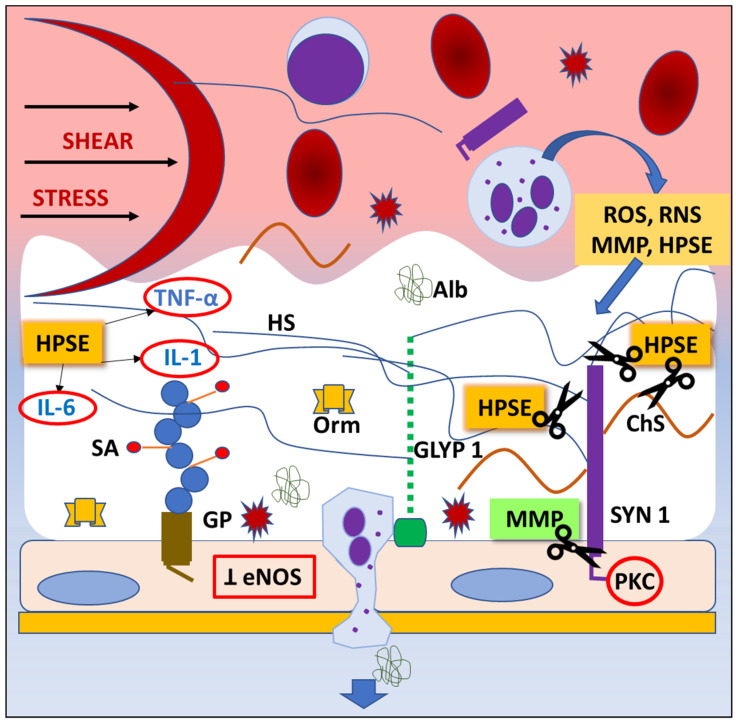
Mechanisms of endothelial glycocalyx degradation (“shedding”). Any pathological situation, like trauma or ischemia/reperfusion injury, can lead to degradation of endothelial glycocalyx (EG). Shear stress activates inflammatory cells, which release highly reactive chemicals (ROS, RNS), cytokines, and proteaze enzymes. Consequently, the inhibition of endothelial nitric oxide synthase (eNOS) synthesis and protein kinase C (PKC) activity result in impared ability of vasodilatation and inhibition of intracellular signaling pathways, thus leading to EG degradation, dysfunction of endothelial cells’ regulatory functions, and leukocyte and platelet adhesion. Abbreviations: Alb—albumin, ChS—chondroitin sulphate, eNOS—endothelial nitric oxyde synthase, GLYP 1—glypican 1, GP—glycoprotein, HPSE—heparanase, HS—heparan sulphate, IL-1—interleukin-1, IL-6—interleukin-6, MMP—matrix metaloproteinase, Orm—orosomucoid, PKC—protein kinase C, RNS—reactive nitrogen species, ROS—reactive oxygen species, SA—sialic acid, SYN 1—syndecan 1, TNF- α—tumor necrosis factor alpha.

Since the endothelial glycocalyx covers the luminal side of the blood vessels [36], it participates in the regulation of endothelial permeability and leukocyte and platelet adhesion [37,38,39]. Thus, it contributes to the physical and biochemical health of the endothelium and the vasculature [40,41,42,43]. Additionally, the endothelial glycocalyx is constantly exposed to the circulating enzymes, which cause the mechanical and biochemical degradation of the endothelial glycocalyx followed by the renovation process [44], making it a very dynamic structure (Figure 2).

## 3. Endothelial Glycocalyx Shedding in Cardiac Surgery

The endothelial glycocalyx is a dynamic structure characterized by an ongoing balance between its degradation and restoration, which is a result of continuous exposure to pressure and shear stress. Trauma, ischemia/reperfusion injury, and the contact of blood with the artificial surface of the CPB circuit contribute to acute inflammation during cardiac surgery, which can lead to the degradation (“shedding”) of the endothelial glycocalyx.

Inflammatory response and surgical trauma lead to an activation of the immune response, predominantly cytokine and chemokine production, complement activation, and production of reactive oxygen species (ROS) and reactive nitrogen species (RNS) [15,45]. Activated neutrophils and mast cells produce RNS and ROS and release a variety of enzymes, predominantly heparanases, hyaluronidases, neuraminidases, metalloproteinases, and matrix metalloproteinases (MMPs) that shed glycocalyx components [46,47]. When released, heparanase cleaves heparan sulphate and facilitates glycocalyx degradation [48]. In addition, heparanase mediates the release of proinflammatory cytokines, including tumor necrosis factor-alpha (TNF-α), IL-1 and IL-6, interferon-gamma, and chemokines (CXCL-8) embedded in the glycocalyx layer [49] that have been shown to further mediate glycocalyx shedding [50]. Furthermore, proinflammatory cytokines promote phagocytes to release MMPs, which shed components of the endothelial glycocalyx including glypican-1 and endomucin [51]. Diminished concentrations of glypican-1 and endomucin promote leukocyte binding to endothelium, leading to impaired mechanosensation [52,53].

It seems that one of the main roles in endothelial glycocalyx shedding has a metalloproteinase protein family [54,55]. Extracellular matrix turnover is regulated by MMPs, which are a family of endogenous proteolytic enzymes, containing zinc responsible for the degradation of the extracellular matrix and capable of degrading endothelial cell-surface proteins. Consequently, these processes lead to an oxidative stress-induced disruption of the endothelial glycocalyx. Being synthesized by endothelial cells, MMPs play a central role in vascular remodeling. Increasing scientific evidence shows that MMP dysregulation is essential in cardiovascular pathologies [56]. Atherosclerotic plaques are known to show higher expressions of MMP-1, -2, and -9, and patients with ischemic heart disease have higher levels of circulating MMPs than healthy controls, suggesting that matrix remodeling via MMPs may represent an important therapeutic target [44,57,58]. In this context, Ali et al. [44] have shown that histone deacetylase represents a novel epigenetic regulatory mechanism in the oxidative stress-mediated MMPs’ upregulation and their tissue inhibitor downregulation, leading to glycocalyx remodeling and highlighting a potential novel therapeutic target.

Activated MMPs, especially MMP-9 and MMP-13, can cleave syndecan-1 below the attachment site, thereby inducing the shedding of syndecan-1 and consequently of hyaluronic acid and chondroitin sulphate, which are attached to syndecan-1 [15,59].

It has been shown that MMPs cleave hyaluronan receptor CD44, resulting in additional glycocalyx damage [52]. When shed, the increased plasma concentration of glycocalyx components can be found in the plasma and easily detected [60]. Recently, increases in syndecan-1, heparan sulphate, and hyaluronan were detected in patients undergoing cardiac surgery [61]. Shed glycocalyx components promote a vicious cycle that increases the production of ROS and RNS, which further damage the endothelial glycocalyx. In health, the production of ROS and RNS is constantly reduced by the antioxidant system, but in a pathological state such as cardiac surgery, the imbalance occurs in favor of oxidation, which promotes proteolysis and glycocalyx shedding, and increases vascular permeability and endothelial dysfunction [62]. Endothelial degradation mediated by ROS and RNS can occur directly by the activation of enzymes that shed components of the endothelial glycocalyx, predominantly chondroitin, heparan sulfate, dermatan sulfate, and hyaluronic acid [63,64], or indirectly via activation of MMPs and inhibition of the endogenous antioxidant system [47,65].

In addition to endothelial degradation caused by inflammation, glycocalyx shedding during cardiac surgery can be a result of ischemia-reperfusion injury [66]. Cardiac ischemia-reperfusion injury occurs during percutaneous coronary angioplasty, CABG, and heart transplant surgery [67]. Studies have shown that cardiac surgery and CPB could result in the degradation of the glycocalyx and the shedding of its components, such as syndecan-1 and heparan sulfate, into the bloodstream [68,69,70,71,72].

Recently, Dekker et al. [61], as well as some previous studies [61,73,74], have shown that CPB-associated microcirculatory perfusion disturbances persist for the first three postoperative days, underlining the fragility of the microvascular network and delayed restoration capacity following acute injury.

A recent study elucidated the impact of the ischemia-reperfusion phenomenon on glycocalyx degradation during early reperfusion in clinical open-heart surgery. Aortic declamping provoked the rapid elevation of systemic levels of extracardiac syndecan-1. Syndecan-1 concentrations in systemic circulation began to increase already before aortic cross-clamping, that is, before the onset of ischemia [71,75]. Findings from the animal model [76] showed that ischemia-reperfusion injury can shed the endothelial glycocalyx via increased production of ROS and RNS or secondary inflammatory response [77,78], resulting in increased serum concentrations of syndecan-1 and heparan sulphate [75,77].

The use of CPB also induces an intense inflammatory response initiated by the contact of blood with an artificial foreign surface and cardioplegia delivery [77]. Interestingly, high concentrations of syndecan-1 and heparan sulphate are found in patients undergoing off-pump CABG surgery despite the lack of CPB. This can result from ischemia-reperfusion injury from the temporary ligation of coronary arteries, reversible low cardiac output during surgery, or hypotension [76].

The administration of fluids to the patients before the induction of anesthesia is a common practice based on the preoperative assumption of volume depletion because of fluid shifting and blood loss during surgery, and due to perioperative fasting. Accumulating evidence is against that practice, showing that infused fluids can cause the elevation of biomarkers suggestive of endothelial glycocalyx shedding [72,79]. A recent study showed a consistent increase in heparan sulfate for each liter of intravenous fluid delivered, suggesting that fluid resuscitation could lead to iatrogenic damage to the endothelium, resulting in a poor outcome and increased mortality [80]. To avoid acute volume loading that can lead to excessive hemodilution, increased microvascular permeability and edema, researchers suggest using rational fluid management during surgery, matching the type and volume of the fluids to the patient’s actual clinical needs [72,81].

The animal studies showed that it takes five to seven days for the endothelial glycocalyx to restore to its native thickness after shedding [55]. However, preclinical and clinical studies in humans showed a more rapid restoration of the damaged endothelial glycocalyx. In their experimental study, Menash et al. showed rapid adherence of heparan sulfate to the damaged glycocalyx, while more recently, the measurement of glycocalyx by in vitro microscopy revealed the thickening of the damaged endothelial glycocalyx in the clinical setting [82].

## 4. The Impact of Endothelial Glycocalyx Shedding on Endothelial Cells in Cardiac Surgery

There are many physiological functions of the endothelium, and the estimated weight of endothelial cells in an adult is about 1 kg, so we can consider the endothelium to be an organ [82]. The endothelium provides a structural barrier between the blood and solid tissues and monitors the flow of nutrients, diffusion of oxygen, carbon dioxide, and hydrogen ions, and transport of hormones and regulatory molecules [83,84]. Endothelial cells regulate blood vessel tone, local tissue flow, and in the long term, the density of the blood vessels in tissue by paracrine and endocrine effects [85]. The endothelial glycocalyx is situated on the surface of endothelial cells and maintains smoothness and reduces friction to the blood flow, thus supporting the normal function of the endothelium in the supervision of the clothing process, fibrinolysis, and leukodiapedesis [43,86]. This complex physiological role of the endothelium is altered under the influence of harmful substances and inflammatory mediators [83]. Various stimuli and risk factors stimulate endothelial cells to produce and secrete cytokines, chemokines, and growth factors into circulation [87].

Endothelial cells express mechanoreceptors, which allow them to sense changes in blood flow [88]. In areas of low or oscillation shear stress, turbulent blood movement damages the glycocalyx and leaves the surface of endothelial cells unprotected [89,90,91]. The stripped endothelial cells become dysfunctional and lose their fine physiological regulatory functions, such as flow-dependent vasodilatation and capillary-level barrier function, and acquire adhesive properties towards leukocytes and platelets, thus initiating the soft plaque formation that becomes prone to rupture over time [87,91,92,93]. In the setting of shear stress such as CPB, the endothelial glycocalyx degradation products and proteases [93] were found in the circulation compared with healthy controls, and they particularly increased during ischemia [93]. Following cardiac surgery, elevated levels of degraded glycocalyx components such as heparan sulphate, syndecan-1, and hyaluronan are detected in the blood and urine of patients due to the activation of sheddases, heparinase, MMPs, and hyaluronidase, respectively, which are all probably of endothelial origin [87,91,93,94]. Additionally, the atrial natriuretic peptide can shed hyaluronic acid and syndecan-1, and there are other possible sheddases, such as thrombin, elastase, plasmin, tryptase, and cathepsin B [93]. To date, it is not known whether endothelial glycocalyx degradation products could stimulate circulating antigen-presenting cells comprising circulating endothelial cells and emphasize their antigen-presenting properties that support and direct the immune response of T cells. It is of scientific interest that the memory T cell subset provides a sustained immune response in patients during and after cardiac surgery [95,96]. Active cytotoxic Th1 lymphocytes rich in the cytotoxic mediator TNF-α receptor apoptosis-induced ligand are considered responsible for the damage to the endothelial cells [97,98]. It is not known whether the concentration of circulating endothelial cells correlates with glycocalyx degradation products in patients during and after CPB, although damage to the endothelial glycocalyx leads to the detachment of the dysfunctional endothelial cells from the basement membrane [99], increased frequency of dysfunctional circulating endothelial cells and memory T cells [100,101]. There may be a correlation between endothelial glycocalyx degradation products and the frequency of circulating endothelial cells and memory T cells, as they are all increased in the exacerbation of endothelial dysfunction during CPB. Circulating endothelial cells could act as non-professional antigen-presenting cells and they could recognize, bind, process, and present glycocalyx degradation products originating from the damaged arterial wall to the memory effector T lymphocytes in direct contact during CPB [102]. Production of a particular set of cytokines by activated circulating endothelial cells could provide a specific microenvironment, which supports the pro-inflammatory orientation of the immune response in patients during and after CPB. Therefore, plasma concentrations of endothelial glycocalyx components and proteases could be biological laboratory markers of endothelial cell damage and activation of dysfunctional endothelium during CBP, which might govern the pro-inflammatory immune system activation and can be a useful prognostic tool. In experiments in vitro, glycocalyx degradation products are recognized by pattern recognition receptors (PRRs) on the surface of human dendritic cells, human monocyte-derived macrophages, human T and B lymphocytes, NK cells, and endothelial cells as danger signals [103,104,105]. Heparan sulphate and small fragments of hyaluronic acid bound to toll-like receptors TLR-2 and TLR-4 eventually lead to the activation of transcription factors such as NFkB, JNK, and AP-1, and subsequent induction of genes important in immune response [95,106]. The result is the increased synthesis of cytokines TNF-α, IL-1β, IL-2, myocardial level of IL-6, and chemokines MIP-2, KC, RANTES, and MCP-1 [95,106]. Small hyaluronic fragments have this effect in endothelial cells, dendritic cells, macrophages, fibroblasts, and epithelial cells [106]. In this setting, TLR signaling leads to the activation of T cells, vascular dysfunction, and ischemia-reperfusion injury [106,107,108,109].

Endothelial cells in mice express TLR-2 whose endogenous ligands are biglycan and hyaluronic acid fragments as well as TLR-4 with its known ligands like oxidized LDL and HSP60 [110]. In vitro, TLR-2 and TLR-4 expressed on macrophages after stimulation support a pro-inflammatory immune response by the production of TNF-α and MIP-2 [111]. Accordingly, in vivo, the activation of TLR-2 and TLR-4 in mice shows a proatherogenic effect [112]. Human vascular endothelium expresses PRRs, among which TLR-1, TLR-2, TLR-4, and TLR-6 are mainly located in the aorta, carotid and subclavian artery, and temporal, mesenteric, and iliac arteries; while TLR-3 is located in the aorta, and TLR-7 and TLR-9 are located in iliac arteries and increased during endothelial dysfunction underlying arterial hypertension, diabetes, hypercholesterolemia, and hyperuricemia, which ensure endothelial cell activation with PRR ligands [112,113,114,115].

Human-circulating endothelial cells express a wide range of co-stimulatory molecules in different amounts, such as CD80, CD86, OX40, ICOS, CD137, CD2, and CD58, which enable them to provide the second signal for specific activation of memory T cells after binding to corresponding receptors such as ICOS, 4-1BB (CD137), and OX40 on T cells, respectively [111,115].

Endothelial cells are sentinels of local tissue antigens, including degraded glycocalyx components, which are able to recognize and present them to the circulating effector memory cells, serving as non-professional antigen-presenting cells [116]. However, endothelial cells seem unable to provide adequate co-stimulation for naïve T cells to promote their proliferation, cytotoxic potential, and Th1 differentiation, but can stimulate them to produce cytokines [110,116].

## 5. Detection of Endothelial Glycocalyx Shedding in Cardiac Surgery

Standard laboratory and biochemical techniques are widely used in scientific and clinical investigations to evaluate soluble glycocalyx shedding parameters in cardiac surgery. Knowledge of the concentration changes of circulating molecules involved in endothelial glycocalyx shedding is essential for understanding the mechanisms of these processes and developing new therapeutic strategies. Most of the crucial bioactive molecules, metabolites, cytokines, and other parameters included in endothelial glycocalyx shedding in cardiac surgery can be detected, as well as their concentrations measured in serum or plasma by commercially available enzyme-linked immunosorbent assay (ELISA) or enzyme immunoassay (EIA) kits, following manufacturer’s protocols. The most studied endothelial glycocalyx biomarkers in cardiac surgery and their changes are shown in Table 1 [12,61,70,75,117,118,119,120,121,122,123,124,125,126,127,128,129,130,131,132,133,134,135].

The protein expression of target peptides/proteins can be quantified by the Western blot technique. However, to identify novel microvascular variables related to the level of microvascular dysfunction, an effective method including sublingual videomicroscopy by sidestream darkfield, including the Gycocheck™ software imaging, has been developed [136]. This technique allows us to evaluate endothelial surface layer properties and microvascular perfusion, quantify vascular density, perfused boundary region correlated with endothelial glycocalyx dimensions, red blood cell content and velocity, as well as blood flow in sublingual microvessels, absolute and static capillary blood volume, capillary recruitment and dynamic capillary blood volume, and other parameters, which can be used to assess and associate the microvascular health score with disease severity [137]. Knowledge of the interdependence between these variables is the key to understanding microvascular dysfunction, and this method has a high potential to detect microvascular dysfunction in critically ill patients.

## 6. Strategies to Protect and Restore Endothelial Glycocalyx in Cardiac Surgery

Possibilities to protect and regenerate the glycocalyx are the subject of intensive research [138,139,140,141,142,143,144]. There is accumulating evidence that meticulous fluid therapy, volatile anesthesia, normoglycemia, and maintenance of normal plasma albumin levels can minimize glycocalyx injury. The regeneration and protection of the glycocalyx are especially significant in the early perioperative period.

### 6.1. Fluid and Volume Management and Protein-Based Therapy

It has been suggested that during sepsis there is a dissociation between the macrocirculatory and microcirculatory systems. The levels of endothelial glycocalyx shedding and microcirculatory disorder do not always coincide, and the infused fluids may cause an increase in biomarkers indicative of detachment of the endothelial glycocalyx [143,144,145]. A recent study showed a consistent increase in heparan sulfate for each liter of intravenous fluid delivered, suggesting that fluid resuscitation could lead to iatrogenic damage to the endothelium [146]. Similarly, fluid management is important for a cardiac surgery patient in the perioperative period since acute hypervolemic hemodilution could result in mechanical stress and natriuretic peptide-mediated glycocalyx injury, leading to loss of administered fluids in the interstitial space [147]. Hypervolemia can cause endothelial glycocalyx shedding. Liberal perioperative fluid administration resulting in a positive fluid balance is associated with increased morbidity [79]. The main principles guiding fluid therapy consider a zero-balance approach in all patients, maintaining central euvolemia and avoiding hypervolemia in the perioperative period. Glycocalyx-sparing “restrictive” fluid regimens have been shown to reduce postoperative morbidity and lengthy hospitalization, compared with “liberal” regimens [79,148,149].

Human albumin and fresh frozen plasma are blood products that could be applied for glycocalyx regeneration [150]. Fresh frozen plasma contains all plasma proteins needed for the regeneration of the endothelial glycocalyx with strong protective and regenerative effects [150,151]. It has been shown that treatment with fresh frozen plasma results in an increase in glycocalyx thickness and an increased level of syndecan-1 following hemorrhage [144]. It has been suggested that the positive effects of fresh frozen plasma on the endothelial glycocalyx could partly be attributed to fibrinogen, but further studies are needed to elucidate the therapeutic potential of fibrinogen [152,153]. Human albumin is the standard treatment in cases of low blood volume and water retention, and it has been suggested to have protective and restoration effects on the endothelial glycocalyx, physically reinforcing its structure and overweighting the shedding, thus ensuring the integrity of endothelial monolayer through the transport of sphingosine-1-phosphate [154,155,156,157,158]. Beneficial effects of albumin bound to the glycocalyx have been demonstrated in rats and guinea pigs [156,159,160,161,162,163,164], but there are conflicting results with human albumin when compared to fresh frozen plasma [165,166]. In addition, in an animal model of heart transplantation, supplementing histidinetryptophan-ketoglutarate solution with 1% human albumin reduced glycocalyx shedding, myocardial oedema, and intracoronary adhesion of leukocytes [159]. In hemorrhagic shock, plasma replacement improves glycocalyx parameters compared to resuscitation with lactated Ringer’s solution [142].

### 6.2. Maintaining Normoglycemia

Both acute and chronic hyperglycemia have been suggested to cause glycocalyx damage and cardiovascular complications, which are major causes of mortality in patients with diabetes mellitus [163]. In vitro studies with endothelial cells showed that high-glucose media significantly decreases glycosaminoglycan, syndecan-1 and heparan sulphate levels. The loss of heparan sulphate results in endothelial dysfunction because of the increased permeability and impaired NO production [164] In vivo study showed that acute hypergycemia resulted in a 50% glycocalyx thickness reduction [165]. Therefore, maintaining normoglycemia is important in the perioperative period since even brief exposure to hyperglycemia could result in glycocalyx shedding [166,167]. In addition, O’Hora et al. reported that both insulin and metformin increased arterial dilatation with a direct effect on NO synthesis in anesthetized pigs [168].

Empagliflozin, a sodium–glucose co-transporter-2 inhibitor (SGLT2i), has been found to preserve glycocalyx integrity, increase heparan sulphate synthesis, and restore the mechanotransduction response of endothelial cells with damaged glycocalyx in human abdominal aortic endothelial cells [169].

Metformin is a biguanide antidiabetic drug that lowers glucose levels and has been suggested to have protective effects against vascular complications [136,170,171]. Metformin treatment has been shown to reduce the frequency of major adverse cardiac events after 24 months of follow-up in prediabetic patients [172]. It has been shown to counter the effect of hyperglycemia and enhance glycocalyx density and thickness in association with the effect on glycocalyx function and reduction of hyperglycemia-induced endothelial cell surface adhesion molecules E-selectin and intracellular adhesion molecule-1 (ICAM-1) [141]. It has been found to decrease blood concentrations of inflammation markers such as IL-1, IL-6, TNF-*α*, and CRP in prediabetic patients with stable angina and non-obstructive coronary stenosis [173]. In addition, metformin has also been shown to reduce glycocalyx-dependent stiffness and actin polymerization [141]. In metformin-treated male diabetic mice, there was an increase in whole-body glycocalyx volume similar to that of non-diabetic control [140]. We must keep in mind the potential risk of metformin usage in patients with unstable or acute cardiovascular conditions [174,175].

### 6.3. Atherosclerotic Plaque Stabilizers

Sulodexide has been suggested as the most suitable for glycocalyx regeneration as it is a source of the glycocalyx constituent heparan sulphate [176,177,178]. It is used in the treatment of peripheral vascular diseases such as diabetic nephropathy and prophylaxis and in the treatment of thromboembolic diseases [176]. Treatment with sulodexide in patients with type 2 diabetes mellitus during a 2-month period has been shown to increase glycocalyx thickness in retinal and sublingual microcirculation and reduce the transcapillary escape rate of albumin [177]. Sulodexide has been shown to improve proteinuria in patients with diabetes mellitus [179,180], which is an indirect marker of glycocalyx destruction [181,182] and microvascular dysfunction due to increased permeability [183,184]. Due to competitive binding to glycocalyx-associated proteins, the heparin portion of sulodexide could result in heparan sulfate glycocalyx component shedding [185,186]. In addition, dermatan sulfate is not a natural component of the endothelial glycocalyx, and as such, could be considered a foreign substance [150]. There is no evidence that sulodexide could restore glycocalyx-mediated endothelial cell function and interendothelial communication as in healthy vessels [40]. Sulodexide exerts minimal anti-coagulant effects in vivo, but attention is necessary when administered in larger doses in the perioperative setting due to a potential bleeding tendency [187]. Recent evidence suggests that statins may prevent endothelial dysfunction by decreasing the expression of adhesion and inflammation molecules. A low simvastatin dose has been shown to reduce the expression of VCAM-1 and ICAM-1. In addition, statin therapy inhibited endothelial reticulum stress by reducing intracellular cholesterol accumulation and by blocking intracellular signal transduction [188]. The statin representative, rosuvastatin, has been shown to significantly increase endothelial cell glycocalyx volume in patients on hypercholesterolemia therapy but not to non-cholesterolemic levels [189]. Statin therapy has been suggested as a promising perioperative intervention because short-term administration of rosuvastatin to patients with familial hypercholesterolemia improved systemic glycocalyx volume [189]. Although rosuvastatin appears to increase capillary glycocalyx thickness in hypercholesterolemic patients, it did not decrease glycocalyx permeability in hypercholesterolemia, suggesting limitations in glycocalyx regeneration [189].

### 6.4. Anti-Inflammatory Treatment

Inflammatory stimuli increase the production of pro-inflammatory mediators such as ROS, RNS, and pro-inflammatory cytokines that activate MMS able to cleave proteoglycans, thus degrading the glycocalyx [190,191]. Blocking the proinflammatory cascade could reduce glycocalyx degradation [191]. Increased glycocalyx shedding associated with increased plasma levels of syndecan, heparan sulphate, and hyaluronan has been detected in septic patients in correlation with severity [192,193,194]. One should keep in mind that therapeutic approaches that decrease the immune response can render the patient more susceptible to infection. Etanercept, a TNF-α inhibitor, has been shown to reduce inflammation-induced glycocalyx shedding [195,196]. In the study by Nieuwdorp et al., healthy adult volunteers received a low-dose endotoxin to induce glycocalyx destruction, and it was found that treatment with etanercept abolished the elevation in endotoxin-induced elevation of plasma levels of hyaluronic acid and hyaluronidase, as well as limited the endotoxin-induced reduction in glycocalyx thickness, although glycocalyx thickness did not reach pre-endotoxin levels [197]. In the study by Chappell et al., pretreatment with hydrocortisone has been found to alleviate glycocalyx destruction caused by inflammation due to infused TNF-α or ischemia in guinea pig hearts [198]. In addition, hydrocortisone reduced glycocalyx degradation in ischemia-reperfusion models [66], and completely suppressed the shedding of syndecan-1 and heparan sulfate in inflammatory conditions caused by TNF-α [198]. Stress doses of hydrocortisone have been found to attenuate perioperative inflammatory responses and improve early postoperative outcomes in high-risk cardiac surgery patients [199,200]. Brettner et al. showed that pretreatment with hydrocortisone ameliorated the shedding of heparan sulfate in patients undergoing cardiac surgery with CPB but had no relevant influence on various clinical parameters or patient mortality [129]. Hydrocortisone is also the recommended treatment in patients with septic shock refractory to fluids and vasopressors [201].

The anti-inflammatory drug poloxamer-188 used to increase tissue oxygenation and reduce painful episodes in sickle cell disease has been suggested to have a beneficial effect on endothelial cell glycocalyx [202,203]. In the study performed on Sprague-Dawley male rats, Torres et al. have found that post-hemorrhagic treatment with poloxamer-188 could restore glycocalyx thickness to 85% of pre-hemorrhagic baseline conditions, significantly lower plasma syndecan-1 level, and decrease glycocalyx-associated vascular permeability to the pre-hemorrhagic baseline level [203]. Since atherosclerosis is considered a chronic inflammatory disease, it has been suggested that novel anti-inflammatory drugs may be useful to prevent endothelial dysfunction and coronary artery disease. Tocilizumab is a monoclonal human antibody that blocks IL-6 receptors and is used in the treatment of rheumatoid arthritis. In a recent study on patients with rheumatoid arthritis, tocilizumab treatment was found to increase endothelial glycocalyx thickness and reduce arterial stiffness [204]. The tyrosine-kinase inhibitor imatinib was found to ameliorate endothelial dysfunction in rabbits on a high cholesterol diet. In addition, imatinib treatment decreased blood CRP and lipid levels [205].

### 6.5. Anticoagulants

Anticoagulant antithrombin supplementation has been widely used to treat sepsis-induced disseminated intravascular coagulation. A multicenter retrospective study suggested a trend towards reduced in-hospital all-cause mortality in patients receiving antithrombin supplementation [206]. In the study by Chappell et al., antithrombin treatment has been found to alleviate glycocalyx destruction, suppress shedding of syndecan-1 and heparan sulfate, and block vessel leakage caused by inflammation due to infused TNF-α or ischemia in guinea pig hearts [198,207]. El Saadani et al. showed that intravenous antithrombin and enoxaparin treatment reduced leukocyte adhesion and transit in the blood–brain barrier after traumatic brain injury, suggesting possible usage in the restoration of glycocalyx barrier functionality [208]. Lipowski et al. showed that low-molecular-weight heparin can inhibit glycocalyx shedding and decrease leukocyte adhesion in male Wistar rats [185]. In a septic shock model, Yini et al. showed that treatment with crystalloids and antibiotics only partially reversed the glycocalyx degradation, while the treatment addition of unfractionated heparin normalized the endothelial glycocalyx, suggesting a protective or anti-inflammatory effect of heparin leading to reduced glycocalyx shedding [209]. On the other hand, VanTeeffelen et al. showed that heparin competed with the heparan sulfate component of the glycocalyx, releasing the proteins bound to heparan sulfate, thus leading to the degradation of the glycocalyx structure and impairment of its barrier function [210]. Intravenous injection of low-molecular heparin results in a 3-fold increase in the enzymatic activity responsible for the release of embedded proteins from endothelial cell glycocalyx and increased protein detachment from the glycocalyx into the plasma in diabetic patients and control [211,212]. Thus, the role of heparin in the protection and regeneration of the endothelial glycocalyx is controversial, and although heparin could prevent endothelial glycocalyx shedding, further studies are needed to elucidate a potentially negative effect on barrier functionality [185,211,212].

### 6.6. Anaesthetics and Anesthetic Technique

There is cumulating experimental evidence that volatile anesthetics such as sevoflurane have been found to protect the glycocalyx in post-ischemic coronary beds and improve coronary vascular function [39,213]. There are experimental studies on the protective effect of sevoflurane against endothelial glycocalyx degradation by ischemia-reperfusion injury, and it has been shown to protect the endothelial glycocalyx better than propofol against ischemia-reperfusion injury in a porcine model [39,213,214]. In contrast to the experimental results, sevoflurane did not show a better protective effect on the endothelial glycocalyx than propofol in clinical studies of lung resection and knee ligament surgery [215,216]. A recent study conducted in patients with gastric cancer undergoing minimally invasive gastrectomy demonstrated that total intravenous anesthesia with propofol and remifentanil showed superior protective effects against endothelial glycocalyx damage during surgery in contrast to volatile anesthesia with sevoflurane and remifentanil. Both types of anesthetics could not prevent postoperative syndecan-1 shedding, supporting the previous clinical studies showing comparable effects of these agents on endothelial glycocalyx damage in surgical patients [217]. A recent randomized control study demonstrated that sevoflurane could decrease glycocalyx degradation in patients undergoing heart valve surgery with CPB [218].

Epidural anesthesia may partially attenuate the surgical inflammatory response, and it is conceivable that glycocalyx would benefit from such practice. Single-shot neuraxial anesthesia, however, does not appear to dampen the inflammatory response to the same degree [219,220,221].

## 7. Conclusions

Cardiac surgery is one of the highest-risk procedures because it results in endothelial damage, which contributes to the development of organ dysfunction in the perioperative period. Understanding the physiology and pathophysiology of many aspects of the endothelial glycocalyx allows clinicians to choose the most appropriate technique and fluid management that can help prevent glycocalyx damage or degradation during cardiac surgery. It has been shown that endothelial glycocylyx is disrupted at the early stages of disease development, suggesting that glycocalyx components may be potential biomarkers of early disease. However, despite the known pathological sequences at the molecular level that lead to glycocalyx damage, with serious consequences for patients undergoing cardiac surgery, the clinical utility is unfortunately limited to meticulous fluid management, normoglycemia maintenance, and albumin use, which can help prevent glycocalyx damage or degradation during cardiac surgery. Therefore, it is critical to develop new approaches and therapeutic strategies targeted at the protection and restoration of the glycocalyx. Further scientific efforts should be invested in recognizing key events behind glycocalyx injury for the purpose of discovering new therapeutic options for endothelial glycocalyx protection in cardiovascular pathologies and cardiovascular surgery.

## Figures and Tables

**Table 1:** The most commonly studied endothelial glycocalyx biomarkers and their changes in patients undergoing cardiac surgeries.

Endothelial Disfunction Marker	Mechanism of Action	Type of Procedure/ Pathology	Biomaterial	Change	Reference
Heparan sulphate	Regulates cell growth, inflammatory response, blood coagulation process	CABG, AVR	S, P	↑ 0 ↓	[70,75,129,131,134] [128] [61,130]
Syndecan-1	Growth factor receptor activation, cell adhesion, matrix adhesion	CABG, AVR	S, P	↑ 0	[12,70,75,128,129,130,131,133,134] [61]
Hyaluronic acid	lymphocyte activation, tissue regeneration, inflammation response, angiogenesis	CABG	S, P, U	↑	[70,129,130,131,132,134]
Chondroitin sulphate	Tissue elasticity	CABG	S	↑	[122]
Soluble vonWillebrand Factor Antigen (vWFAg)	Platelet adherence	CABG, AVR	P	↑	[119,126,127]
E-selectin	Cell adhesion	OPCABG, CABG	P	↑ 0	[121,131,135] [117]
P-selectin	Cell adhesion	OPCABG, CABG	P	↑	[124,135]
ICAM-1	Cell to cell, cell to extracellular matrix adhesion	OPCABG, CABG	P	↑	[117,124,135]
VCAM-1	Cell to cell, cell to extracellular matrix adhesion	OPCABG, CABG	P	↑	[117,121]
VEGF	Cell to cell, cell to extracellular matrix adhesion	OPCABG, CABG	P	↑	[125]
Angiopioetin-1	Angiogenesis, endothelial cell migration, endovascular lining	OPCABG, CABG	P	↓	[120,123]
Angiopioetin-2	Angiogenesis, endothelial cell migration, endovascular lining	OPCABG, CABG	S	↑	[118,120,123,131]

Biomaterial: P—plasma, S—serum, U—urine; Type of procedure/pathology: CABG –coronary artery bypass grafting, OPCABP—off pump coronary artery bypass grafting, AVR—aortic valve replacement; Change: (↑) increase, (↓) decrease, (0) no change.

## References

[1] Verrier E.D. (1999). Cardiac surgery. J. Am. Coll. Surg..

[2] Vervoort D., Meuris B., Meyns B., Verbrugghe P. (2020). Global cardiac surgery: Access to cardiac surgical care around the world. J. Thorac. Cardiovasc. Surg..

[3] Wahba A., Milojevic M., Boer C., De Somer F.M.J.J., Gudbjartsson T., van den Goor J., Jones T.J., Lomivorotov V., Merkle F., Ranucci M. (2020). EACTS/EACTA/EBCP Committee Reviewers. 2019 EACTS/EACTA/EBCP guidelines on cardiopulmonary bypass in adult cardiac surgery. Eur. J. Cardiothorac. Surg..

[4] Siregar S., Groenwold R.H., de Heer F., Bots M.L., van der Graaf Y., van Herwerden L.A. (2012). Performance of the original EuroSCORE. Eur. J. Cardiothorac. Surg..

[5] Libby P., Theroux P. (2005). Pathophysiology of coronary artery disease. Circulation.

[6] Hansson G.K. (2005). Inflammation, atherosclerosis, and coronary artery disease. N Engl. J. Med..

[7] Wenger N.K., Boden W.E. (2010). Ischemic Heart Disease. Institute of Medicine (US) Committee on Social Security Cardiovascular Disability Criteria Cardiovascular Disability: Updating the Social Security Listings.

[8] Stevens J.R., Zamani A., Osborne J.I.A., Zamani R., Akrami M. (2021). Critical evaluation of stents in coronary angioplasty: A systematic review. Biomed. Eng. Online.

[9] Khan M.S., Islam M.Y., Ahmed M.U., Bawany F.I., Khan A., Arshad M.H. (2014). On pump coronary artery bypass graft surgery versus off pump coronary artery bypass graft surgery: A review. Glob. J. Health Sci..

[10] Tan A., Newey C., Falter F. (2022). Pulsatile Perfusion during Cardiopulmonary Bypass: A Literature Review. J. Extra. Corpor. Technol..

[11] Hadi A.R.H., Cornelia S.C., Suwaidi J.A. (2005). Endothelial dysfunction: Cardiovascular risc factors, therapy, and outcome. Vasc. Health Risk Manag..

[12] Pesonen E., Passov A., Andersson S., Suojaranta R., Niemi T., Raivio P., Salmenperä M., Schramko A. (2019). Glycocalyx Degradation and Inflammation in Cardiac Surgery. J. Cardiothorac. Vasc. Anesth..

[13] Song J.W., Goligorsky M.S. (2018). Perioperative implication of the endothelial glycocalyx. Korean J. Anesthesiol..

[14] Krüger-Genge A., Blocki A., Franke R.P., Jung F. (2019). Vascular Endothelial Cell Biology: An Update. Int. J. Mol. Sci..

[15] Foote C.A., Soares R.N., Ramirez-Perez F.I., Ghiarone T., Aroor A., Manrique-Acevedo C., Padilla J., Martinez-Lemus L. (2022). Endothelial Glycocalyx. Compr. Physiol..

[16] Jedlicka J., Becker B.F., Chappell D. (2020). Endothelial Glycocalyx. Crit. Care Clin..

[17] Wang G., Tiemeier G.L., van der Berg B.M., Rabelink T.J. (2020). Endothelial Glycocalyx Hyaluronan: Regulation and Role in Prevention of Diabetic Complications. Am. J. Pathol..

[18] Bennett H.S. (1963). Morphological aspects of extracellular polysaccharides. J. Histochem. Cytochem..

[19] Pillinger N.L., Kam P. (2017). Endothelial glycocalyx: Basic science and clinical implications. Anesth. Intensive Care.

[20] Brouns S.L.N., Provenzale I., van Geffen J.P., van der Meijden P.E.J., Heemskerk J.W.M. (2020). Localized endothelial-based control of platelet aggregation and coagulation under flow: A proof-of-principle vessel-on-a-chip study. J. Thromb. Haemost..

[21] Kincses A., Santa-Maria A.R., Walter F.R., Dér L., Horányi N., Lipka D.V., Valkai S., Deli M.A., Dér A. (2020). A chip device to determine surface charge properties of confluent cell monolayers by measuring streaming potential. Lab. Chip..

[22] Tarbell J.M., Cancel L.M. (2016). The glycocalyx and its significance in human medicine. J. Int. Med..

[23] Cosgun Z.C., Fels B., Kusche-Vihrog K. (2020). Nanomechanics of the endothelial glycocalyx: From structure to function. Am. J. Pathol..

[24] Mahmoud M., Mayer M., Cancel L.M., Bartosch A.M., Mathews R., Tarbell J.M. (2021). The glycocalyx core protein Glypican 1 protects vessel wall endothelial cells from stiffness-mediated dysfunction and disease. Cardiovasc. Res..

[25] Lepedda A.J., Nieddu G., Formato M., Baker M.B., Fernández-Pérez J., Moroni L. (2021). Glycosaminoglycans: From Vascular Physiology to Tissue Engineering Applications. Front. Chem..

[26] Esko J.D., Linhardt R.J., Varki A., Cummings R.D., Esko J.D., Freeze H.H., Stanley P., Bertozzi C.R., Hart G.W., Etzler M.E. (2009). Proteins that bind sulfated glycosaminoglycans. Essentials of Glycobiology.

[27] Gopal S. (2020). Syndecans in Inflammation at a Glance. Front. Immunol..

[28] Zeng Y. (2017). Endothelial glycocalyx as a critical signalling platform integrating the extracellular haemodynamic forces and chemical signalling. J. Cell Mol. Med..

[29] Agere S.A., Kim E.Y., Akhtar N., Ahmed S. (2018). Syndecans in chronic inflammatory and autoimmune diseases: Pathological insights and therapeutic opportunities. J. Cell Physiol..

[30] Pan J., Ho M. (2021). Role of glypican-1 in regulating multiple cellular signaling pathways. Am. J. Physiol. Cell Physiol..

[31] Xie M., Li J.-P. (2019). Heparan sulfate proteoglycan –A common receptor for diverse cytokines. Cell Signal..

[32] Ebong E.E., Lopez-Quintero S.V., Rizzo V., Spray D.C., Tarbell J.M. (2014). Shear-induced endothelial NOS activation and remodeling via heparan sulfate, glypican-1, and syndecan-1. Integr. Biol..

[33] Dragovich M.A., Chester D., Fu B.M., Wu C., Xu Y., Goligorsky M.S., Zhang X.F. (2016). Mechanotransduction of the endothelial glycocalyx mediates nitric oxide production through activation of TRP channels. Am. J. Physiol. Cell Physiol..

[34] Malanovic N., Lohner K. (2016). Gram-positive bacterial cell envelopes: The impact on the activity of antimicrobial peptides. Biochim. Biophys. Acta..

[35] Michen B., Graule T. (2010). Isoelectric points of viruses. J. Appl. Microbiol..

[36] Nieuwdorp M., Meuwese M.C., Vink H., Hoekstra J.B.L., Kastelein J.J.P., Stroes E.S.G. (2005). The endothelial glycocalyx: A potential barrier between health and vascular disease. Curr. Opin. Lipidol..

[37] Weinbaum S., Tarbell J.M., Damiano E.R. (2007). The structure and function of the endothelial glycocalyx layer. Annu. Rev. Biomed. Eng..

[38] Mulivor A.W., Lipowsky H.H. (2002). Role of glycocalyx in leukocyte-endothelial cell adhesion. Am. J. Physiol. Heart Circ. Physiol..

[39] Chappell D., Heindl B., Jacob M., Annecke T., Chen C., Rehm M., Conzen P., Becker B.F. (2011). Sevoflurane reduces leukocyte and platelet adhesion after ischemia-reperfusion by protecting the endothelial glycocalyx. Anesthesiology.

[40] Mitra R., O’Neil G.L., Harding I.C., Cheng M.J., Mensah S.A., Ebong E.E. (2017). Glycocalyx in Atherosclerosis-Relevant Endothelium Function and as a Therapeutic Target. Curr. Atheroscler. Rep..

[41] Davies P.F. (1995). Flow-mediated endothelial mechanotransduction. Physiol. Rev..

[42] Dewey C.F., Bussolari S.R., Gimbrone M.A., Davies P.F. (1981). The dynamic response of vascular endothelial cells to fluid shear stress. J. Biomech. Eng..

[43] Reitsma S., Slaaf D.W., Vink H., van Zandvoort M.A., oude Egbrink M.G.A. (2007). The endothelial glycocalyx: Composition, functions, and visualization. Pflugers. Arch..

[44] Ali M.M., Mahmoud A.M., Le Master E., Levitan I., Phillips S.A. (2019). Role of matrix metalloproteinases and histone deacetylase in oxidative stress-induced degradation of endothelial glycocalyx. Am. J. Physiol. Heart Circ. Physiol..

[45] Ågren M.S., Auf dem Keller U. (2020). Matrix Metalloproteinases: How Much Can They Do?. Int. J. Mol. Sci..

[46] van Golen R.F., van Gulik T.M., Heger M. (2012). Mechanistic overview of reactive species-induced degradation of the endothelial glycocalyx during hepatic ischemia/reperfusion injury. Free Radic. Biol. Med..

[47] Peterson S.B., Liu J. (2013). Multi-faceted substrate specificity of heparanase. Matrix. Biol..

[48] Vlodavsky I., Ilan N., Naggi A., Casu B. (2007). Heparanase: Structure, biological functions, and inhibition by heparin-derived mimetics of heparan sulfate. Curr. Pharm. Des..

[49] Langjahr P., Díaz-Jiménez D., De la Fuente M., Rubio E., Golenbock D., Bronfman F.C., Quera R., González M.J., Hermoso M.A. (2014). Metalloproteinase-dependent TLR2 ectodomain shedding is involved in soluble toll-like receptor 2 (sTLR2) production. PLoS ONE.

[50] Kawahara R., Granato D.C., Yokoo S., Domingues R.R., Trindade D.M., Paes Leme A.F. (2017). Mass spectrometry-based proteomics revealed Glypican-1 as a novel ADAM17 substrate. J. Proteomics..

[51] Yang J., LeBlanc M.E., Cano I., Saez-Torres K.L., Saint-Geniez M., Ng Y.S., D’Amore P.A. (2020). ADAM10 and ADAM17 proteases mediate proinflammatory cytokine-induced and constitutive cleavage of endomucin from the endothelial surface. J. Biol. Chem..

[52] Endo K., Takino T., Miyamori H., Kinsen H., Yoshizaki T., Furukawa M., Sato H. (2003). Cleavage of syndecan-1 by membrane type matrix metalloproteinase-1 stimulates cell migration. J. Biol. Chem..

[53] Strilakou A., Perelas A., Lazaris A., Papavdi A., Karkalousos P., Giannopoulou I., Kriebardis A., Panayiotides I., Liapi C. (2016). Immunohistochemical determination of the extracellular matrix modulation in a rat model of choline-deprived myocardium: The effects of carnitine. Fundam. Clin. Pharmacol..

[54] Cui N., Wang H., Long Y., Su L., Liu D. (2015). Dexamethasone suppressed LPS-induced matrix metalloproteinase and its effect on endothelial glycocalyx shedding. Mediat. Inflamm..

[55] Potter D.R., Jiang J., Damiano E.R. (2009). The recovery time course of the endothelial cell glycocalyx in vivo and its implications in vitro. Circ. Res..

[56] Sun H., Zhang J., Zheng Y., Shang S. (2018). Expressions and clinical significance of factors related to acute coronary syndrome. J. Biol. Regul. Homeost. Agents.

[57] Milusev A., Rieben R., Sorvillo N. (2022). The Endothelial Glycocalyx: A Possible Therapeutic Target in Cardiovascular Disorders. Front. Cardiovasc. Med..

[58] Reine T.M., Lanzalaco F., Kristiansen O., Enget A.R., Satchell S., Jenssen T.G., Kolset S.O. (2019). Matrix metalloproteinase-9 mediated shedding of syndecan-4 in glomerular endothelial cells. Microcirculation.

[59] Sieve I., Münster-Kühnel A.K., Hilfiker-Kleiner D. (2018). Regulation and function of endothelial glycocalyx layer in vascular diseases. Vascul. Pharmacol..

[60] Hahn R.G., Patel V., Dull R.O. (2021). Human glycocalyx shedding: Systematic review and critical appraisal. Acta. Anaesthesiol. Scand..

[61] Dekker N.A.M., Veerhoek D., Koning N.J., van Leeuwen A.L.I., Elbers P.W.G., van den Brom C.E., Vonk A.B.A., Boer C. (2019). Postoperative microcirculatory perfusion and endothelial glycocalyx shedding following cardiac surgery with cardiopulmonary bypass. Anaesthesia.

[62] Jackson-Weaver O., Friedman J.K., Rodriguez L.A., Hoof M.A., Drury R.H., Packer J.T., Smith A., Guidry C., Duchesne J.C. (2019). Hypoxia/reoxygenation decreases endothelial glycocalyx via reactive oxygen species and calcium signaling in a cellular model for shock. J. Trauma. Acute. Care Surg..

[63] Ding Z., Wang X., Khaidakov M., Liu S., Dai Y., Mehta J.L. (2012). Degradation of heparan sulfate proteoglycans enhances oxidized-LDL-mediated autophagy and apoptosis in human endothelial cells. Biochem. Biophys. Res. Commun..

[64] Moseley R., Waddington R.J., Embery G. (1997). Degradation of glycosaminoglycans by reactive oxygen species derived from stimulated polymorphonuclear leukocytes. Biochim. Biophys. Acta..

[65] Wang Y., Herrera A.H., Li Y., Belani K.K., Walcheck B. (2009). Regulation of mature ADAM17 by redox agents for L-selectin shedding. J. Immunol..

[66] Seal J.B., Gewertz B.L. (2005). Vascular dysfunction in ischemia-reperfusion injury. Ann. Vasc. Surg..

[67] Reffelmann T., Kloner R.A. (2006). The no-reflow phenomenon: A basic mechanism of myocardial ischemia and reperfusion. Basic. Res. Cardiol..

[68] Rehm M., Bruegger D., Christ F., Conzen P., Thiel M., Jacob M., Chappell D., Stoeckelhuber M., Welsch U., Reichart B. (2007). Shedding of the endothelial glycocalyx in patients undergoing major vascular surgery with global and regional ischemia. Circulation.

[69] Koning N.J., Vonk A.B.A., Vink H., Boer C. (2016). Side-by-Side Alterations in Glycocalyx Thickness and Perfused Microvascular Density During Acute Microcirculatory Alterations in Cardiac Surgery. Microcirculation.

[70] Wu Q., Gao W., Zhou J., He G., Ye J., Fang F., Luo J., Wang M., Xu H., Wang W. (2019). Correlation between acute degradation of the endothelial glycocalyx and microcirculation dysfunction during cardiopulmonary bypass in cardiac surgery. Microvasc. Res..

[71] Bruegger D., Rehm M., Abicht J., Paul J.O., Stoeckelhuber M., Pfirrmann M., Reichart B., Becker B.F., Christ F. (2009). Shedding of the endothelial glycocalyx during cardiac surgery: On-pump versus off-pump coronary artery bypass graft surgery. J. Thorac. Cardiovasc. Surg..

[72] Svennevig K., Hoel T., Thiara A., Kolset S., Castelheim A., Mollnes T., Brosstad F., Fosse E., Svennevig J. (2008). Syndecan-1 plasma levels during coronary artery bypass surgery with and without cardiopulmonary bypass. Perfusion.

[73] De Backer D., Dubois M.J., Schmartz D., Koch M., Ducart A., Barvais L., Vincent J. (2009). Microcirculatory alterations in cardiac surgery: Effects of cardiopulmonary bypass and anesthesia. Ann. Thorac. Surg..

[74] Cabrales P., Vázquez B.Y., Tsai A.G., Intaglietta M. (2007). Microvascular and capillary perfusion following glycocalyx degradation. J. Appl. Physiol..

[75] Passov A., Schramko A., Salminen U.S., Aittomäki J., Andersson S., Pesonen E. (2021). Endothelial glycocalyx during early reperfusion in patients undergoing cardiac surgery. PLoS ONE.

[76] Chappell D., Bruegger D., Potzel J., Jacob M., Brettner F., Vogeser M., Conzen P., Becker B.F., Rehm M. (2014). Hypervolemia increases release of atrial natriuretic peptide and shedding of the endothelial glycocalyx. Crit. Care.

[77] Mulivor A.W., Lipowsky H.H. (2004). Inflammation- and ischemia-induced shedding of venular glycocalyx. Am. J. Physiol. Heart Circ. Physiol..

[78] Warren O.J., Smith A.J., Alexiou C., Rogers P.L., Jawad N., Vincent C., Darzi A.W., Athanasiou T. (2009). The inflammatory response to cardiopulmonary bypass: Part 1—mechanisms of pathogenesis. J. Cardiothorac. Vasc. Anesth..

[79] Doherty M., Buggy D.J. (2012). Intraoperative fluids: How much is too much?. Br. J. Anaesth..

[80] Myers G.J., Wegner J. (2017). Endothelial Glycocalyx and Cardiopulmonary Bypass. J. Extra. Corpor. Technol..

[81] Schött U., Solomon C., Fries D., Bentzer P. (2016). The endothelial glycocalyx and its disruption, protection and regeneration: A narrative review. Scand. J. Trauma. Resusc. Emerg. Med..

[82] Goncharov N.V., Nadeev A.D., Jenkins R.O., Avdonin P.V. (2017). Markers and Biomarkers of Endothelium: When Something Is Rotten in the State. Oxid. Med. Cell Longev..

[83] Kazmi R.S., Boyce S., Lwaleed B.A. (2015). Homeostasis of Hemostasis: The Role of Endothelium. Semin. Thromb. Hemost..

[84] Pi X., Xie L., Patterson C. (2018). Emerging Roles of Vascular Endothelium in Metabolic Homeostasis. Circ. Res..

[85] Sumpio B.E., Riley J.T., Dardik A. (2002). Cells in focus: Endothelial cell. Int. J. Biochem. Cell Biol..

[86] Gragnano F., Sperlongano S., Golia E., Natale F., Bianchi R., Crisci M., Fimiani F., Pariggiano I., Diana V., Carbone A. (2017). The Role of von Willebrand Factor in Vascular Inflammation: From Pathogenesis to Targeted Therapy. Mediat. Inflamm..

[87] Mai J., Virtue A., Shen J., Wang H., Yang X.F. (2013). An evolving new paradigm: Endothelial cells--conditional innate immune cells. J. Hematol. Oncol..

[88] Gimbrone M.A., García-Cardeña G. (2013). Vascular endothelium, hemodynamics, and the pathobiology of atherosclerosis. Cardiovasc. Pathol..

[89] Alphonsus C.S., Rodseth R.N. (2014). The endothelial glycocalyx: A review of the vascular barrier. Anaesthesia.

[90] Noble M.I., Drake-Holland A.J., Vink H. (2008). Hypothesis: Arterial glycocalyx dysfunction is the first step in the atherothrombotic process. QJM.

[91] Dogné S., Flamion B. (2020). Endothelial Glycocalyx Impairment in Disease: Focus on Hyaluronan Shedding. Am. J. Pathol..

[92] Magoon R., Makhija N. (2020). Endothelial Glycocalyx and Cardiac Surgery: Newer Insights. J. Cardiothorac. Vasc. Anesth..

[93] Becker B.F., Jacob M., Leipert S., Salmon A.H., Chappell D. (2015). Degradation of the endothelial glycocalyx in clinical settings: Searching for the sheddases. Br. J. Clin. Pharmacol..

[94] Raines E.W. (2006). Antigen-independent targeting of long-lived CD4+ cytolytic T effector cells to lesions of atherosclerosis. Circ. Res..

[95] Collins L.E., Troeberg L. (2019). Heparan sulfate as a regulator of inflammation and immunity. J. Leukoc. Biol..

[96] Peng L.P., Cao Y., Zhao S.L., Huang Y.X., Yang K., Huang W. (2019). Memory T cells delay the progression of atherosclerosis via AMPK signaling pathway. Ann. Transl. Med..

[97] Abassi Z., Armaly Z., Heyman S.N. (2020). Glycocalyx Degradation in Ischemia-Reperfusion Injury. Am. J. Pathol..

[98] Kyaw T., Peter K., Li Y., Tipping P., Toh B.H., Bobik A. (2017). Cytotoxic lymphocytes and atherosclerosis: Significance, mechanisms and therapeutic challenges. Br. J. Pharmacol..

[99] Farinacci M., Krahn T., Dinh W., Volk H.D., Düngen H.D., Wagner J., Konen T., von Ahsen O. (2018). Circulating endothelial cells as biomarker for cardiovascular diseases. Res. Pract. Thromb. Haemost..

[100] Mutin M., Canavy I., Blann A., Bory M., Sampol J., Dignat-George F. (1999). Direct evidence of endothelial injury in acute myocardial infarction and unstable angina by demonstration of circulating endothelial cells. Blood.

[101] Hofmann U., Frantz S. (2016). Role of T-cells in myocardial infarction. Eur. Heart J..

[102] Rakic M., Persic V., Kehler T., Bastiancic A.L., Rosovic I., Laskarin G., Sotosek Tokmadzic V. (2018). Possible role of circulating endothelial cells in patients after acute myocardial infarction. Med. Hypotheses.

[103] Muzio M., Bosisio D., Polentarutti N., D’amico G., Stoppacciaro A., Mancinelli R., van’t Veer C., Penton-Rol G., Ruco L.P., Allavena P. (2000). Differential expression and regulation of toll-like receptors (TLR) in human leukocytes: Selective expression of TLR3 in dendritic cells. J. Immunol..

[104] Huebener P., Schwabe R.F. (2013). Regulation of wound healing and organ fibrosis by toll-like receptors. Biochim. Biophys. Acta..

[105] Guo L.Y., Yang F., Peng L.J., Li Y.B., Wang A.P. (2020). CXCL2, a new critical factor and therapeutic target for cardiovascular diseases. Clin. Exp. Hypertens..

[106] Wang Y., Abarbanell A.M., Herrmann J.L., Weil B.R., Poynter J., Manukyan M.C., Crisostomo P.R., Meldrum D.R. (2010). Toll-like receptor signaling pathways and the evidence linking toll-like receptor signaling to cardiac ischemia/reperfusion injury. Shock.

[107] Mehta A.K., Gracias D.T., Croft M. (2018). TNF activity and T cells. Cytokine.

[108] Santarlasci V., Cosmi L., Maggi L., Liotta F., Annunziato F. (2013). IL-1 and T Helper Immune Responses. Front. Immunol..

[109] Huo Y., Weber C., Forlow S.B., Sperandio M., Thatte J., Mack M., Jung S., Littman D.R., Ley K. (2001). The chemokine KC, but not monocyte chemoattractant protein-1, triggers monocyte arrest on early atherosclerotic endothelium. J. Clin. Investig..

[110] Jameson S.C., Masopust D. (2018). Understanding Subset Diversity in T Cell Memory. Immunity.

[111] Pober J.S., Sessa W.C. (2007). Evolving functions of endothelial cells in inflammation. Nat. Rev. Immunol..

[112] Xiao L., Liu Y., Wang N. (2014). New paradigms in inflammatory signaling in vascular endothelial cells. Am. J. Physiol. Heart Circ. Physiol..

[113] Schaefer L., Babelova A., Kiss E., Hausser H.J., Baliova M., Krzyzankova M., Marsche G., Young M.F., Mihalik D., Götte M. (2005). The matrix component biglycan is proinflammatory and signals through Toll-like receptors 4 and 2 in macrophages. J. Clin. Investig..

[114] Wang L., Wang J., Fang J., Zhou H., Liu X., Su S.B. (2015). High glucose induces and activates Toll-like receptor 4 in endothelial cells of diabetic retinopathy. Diabetol. Metab. Syndr..

[115] Pahwa R., Nallasamy P., Jialal I. (2016). Toll-like receptors 2 and 4 mediate hyperglycemia induced macrovascular aortic endothelial cell inflammation and perturbation of the endothelial glycocalyx. J. Diabetes Complicat..

[116] Carman C.V., Martinelli R. (2015). T Lymphocyte-Endothelial Interactions: Emerging Understanding of Trafficking and Antigen-Specific Immunity. Front. Immunol..

[117] Vallely M.P., Bannon P.G., Bayfield M.S., Hughes C.F., Kritharides L. (2010). Endothelial activation after coronary artery bypass surgery: Comparison between on-pump and off-pump techniques. Heart Lung. Circ..

[118] Hadem J., Rossnick R., Hesse B., Herr M., Hansen M., Bergmann A., Kensah G., Maess C., Baraki H., Kümpers P. (2020). Endothelial dysfunction following coronary artery bypass grafting: Influence of patient and procedural factors. Herz.

[119] Kuhn E.W., Choi Y.H., Pyun J.M., Neef K., Liakopoulos O.J., Stamm C., Wittwer T., Wahlers T. (2015). Endothelial Injury Associated with Cold or Warm Blood Cardioplegia during Coronary Artery Bypass Graft Surgery. Biomed. Res. Int..

[120] Hilbert T., Duerr G.D., Hamiko M., Frede S., Rogers L., Baumgarten G., Hoeft A., Velten M. (2016). Endothelial permeability following coronary artery bypass grafting: An observational study on the possible role of angiopoietin imbalance. Crit. Care.

[121] Girão-Silva T., Fonseca-Alaniz M.H., Ribeiro-Silva J.C., Lee J., Patil N.P., Dallan L.A., Baker A.B., Harmsen M.C., Krieger J.E., Miyakawa A.A. (2021). High stretch induces endothelial dysfunction accompanied by oxidative stress and actin remodeling in human saphenous vein endothelial cells. Sci. Rep..

[122] Karangelis D., Asimakopoulou A., Kanakis I., Tagarakis G.I., Koufakis T., Triposkiadis F., Tsilimingas N., Karamanos N.K. (2011). Monitoring serum chondroitin sulfate levels in patients submitted to coronary artery bypass surgery. Biomed. Chromatog..

[123] Parke R., Bihari S., Dixon D.L., Gilder E., Cavallaro E., McGuinness S., Bersten A.D. (2018). Fluid resuscitation associated with elevated angiopoietin-2 and length of mechanical ventilation after cardiac surgery. Crit. Care Resusc..

[124] Serrano C.V., Souza J.A., Lopes N.H., Fernandes J.L., Nicolau J.C., Blotta M.H., Ramires J.A., Hueb W.A. (2010). Reduced expression of systemic proinflammatory and myocardial biomarkers after off-pump versus on-pump coronary artery bypass surgery: A prospective randomized study. J. Crit. Care.

[125] Onorati F., Rubino A.S., Nucera S., Foti D., Sica V., Santini F., Gulletta E., Renzulli A. (2010). Off-pump coronary artery bypass surgery versus standard linear or pulsatile cardiopulmonary bypass: Endothelial activation and inflammatory response. Eur. J. Cardiothorac. Surg..

[126] Lisowska A., Lisowski P., Knapp M., Tycinska A., Sawicki R., Malyszko J., Hirnle T., Musial W.J. (2014). Serum adiponectin and markers of endothelial dysfunction in stable angina pectoris patients undergoing coronary artery bypass grafting (CABG). Adv. Med. Sci..

[127] Hernández-Romero D., Lahoz Á., Roldan V., Jover E., Romero-Aniorte A.I., Martinez C.M., Jara-Rubio R., Arribas J.M., Garcia-Alberola A., Cánovas S. (2016). Von Willebrand factor is associated with atrial fibrillation development in ischaemic patients after cardiac surgery. Europace.

[128] Abou-Arab O., Kamel S., Beyls C., Huette P., Bar S., Lorne E., Galmiche A., Guinot P.G. (2020). Vasoplegia After Cardiac Surgery Is Associated With Endothelial Glycocalyx Alterations. J. Cardiothorac. Vasc. Anesth..

[129] Brettner F., Chappell D., Nebelsiek T., Hauer D., Schelling G., Becker B.F., Rehm M., Weis F. (2019). Preinterventional hydrocortisone sustains the endothelial glycocalyx in cardiac surgery. Clin. Hemorheol. Microcirc..

[130] Hohn A., Baumann A., Pietroschinsky E., Franklin J., Illerhaus A., Buchwald D., Hinkelbein J., Zahn P.K., Annecke T. (2021). Hemoadsorption: Effective in reducing circulating fragments of the endothelial glycocalyx during cardiopulmonary bypass in patients undergoing on-pump cardiac surgery?. Minerva. Anestesiol..

[131] Bol M.E., Huckriede J.B., van de Pas K.G.H., Delhaas T., Lorusso R., Nicolaes G.A.F., Sels J.E.M., van de Poll M.C.G. (2022). Multimodal measurement of glycocalyx degradation during coronary artery bypass grafting. Front. Med..

[132] Zarbock A., Meersch M., Van Aken H., Görlich D., Singbartl K. (2014). Urinary hyaluronic acid as an early predictor of acute kidney injury after cardiac surgery. J. Am. Coll. Cardiol..

[133] Robich M., Ryzhov S., Kacer D., Palmeri M., Peterson S.M., Quinn R.D., Carter D., Sheppard F., Hayes T., Sawyer D.B. (2020). Prolonged Cardiopulmonary Bypass is Associated With Endothelial Glycocalyx Degradation. J. Surg. Res..

[134] Bruegger D., Schwartz L., Chappell D., Jacob M., Rehm M., Vogeser M., Christ F., Reichart B., Becker B.F. (2011). Release of atrial natriuretic peptide precedes shedding of the endothelial glycocalyx equally in patients undergoing on- and off-pump coronary artery bypass surgery. Basic. Res. Cardiol..

[135] Rossaint J., Berger C., Van Aken H., Scheld H.H., Zahn P.K., Rukosujew A., Zarbock A. (2012). Cardiopulmonary bypass during cardiac surgery modulates systemic inflammation by affecting different steps of the leukocyte recruitment cascade. PLoS ONE.

[136] Rovas A., Sackarnd J., Rossaint J., Kampmeier S., Pavenstädt H., Vink H., Kümpers P. (2021). Identification of novel sublingual parameters to analyze and diagnose microvascular dysfunction in sepsis: The NOSTRADAMUS study. Crit. Care.

[137] Fuchs A., Groß S., Neumann T., Illerhaus A., Vink H., Klasen G., Gathof B., Annecke T. (2021). Immediate effects of whole blood donation on the endothelial surface layer and glycocalyx shedding. Blood Transfus..

[138] Mensah S.A., Cheng M.J., Homayoni H., Plouffe B.D., Coury A.J., Ebong E.E. (2017). Regeneration of glycocalyx by heparan sulfate and sphingosine 1-phosphate restores inter-endothelial communication. PLoS ONE.

[139] Giantsos-Adams K.M., Koo A.J.-A., Song S., Sakai J., Sankaran J., Shin J.H., Garcia-Cardena G., Dewey C.F. (2013). Heparan Sulfate Regrowth Profiles Under Laminar Shear Flow Following Enzymatic Degradation. Cell Mol. Bioeng..

[140] Eskens B.J., Zuurbier C.J., van Haare J., Vink H., van Teeffelen J.W. (2013). Effects of two weeks of metformin treatment on wholebody glycocalyx barrier properties in db/db mice. Cardiovasc. Diabetol..

[141] Targosz-Korecka M., Malek-Zietek K.E., Kloska D., Rajfur Z., Stepien E.Ł., Grochot-Przeczek A., Szymonski M. (2020). Metformin attenuates adhesion between cancer and endothelial cells in chronic hyperglycemia by recovery of the endothelial glycocalyx barrier. Biochim. Biophys. Acta. Gen. Subj..

[142] Kozar R.A., Peng Z., Zhang R., Holcomb J.B., Pati S., Park P., Ko T.C., Paredes A. (2011). Plasma restoration of endothelial glycocalyx in a rodent model of hemorrhagic shock. Anesth. Analg..

[143] Long R., Vink H. (2016). (Microvascular Health Solutions LLC). Synergistic Glycocalyx Treatment Compositions and Methods. U.S. Patent.

[144] Torres L.N., Sondeen J.L., Ji L., Dubick M.A., Filho I.T. (2013). Evaluation of resuscitation fluids on endothelial glycocalyx, venular blood flow, and coagulation function after hemorrhagic shock in rats. J. Trauma. Acute. Care Surg..

[145] Rovas A., Seidel L.M., Vink H., Pohlkötter T., Pavenstädt H., Ertmer C., Hessler M., Kümpers P. (2019). Association of sublingual microcirculation parameters and endothelial glycocalyx dimensions in resuscitated sepsis. Crit. Care.

[146] Hippensteel J.A., Uchimido R., Tyler P.D., Burke R.C., Han X., Zhang F., McMurtry S.A., Colbert J.F., Lindsell C.J., Angus D.C. (2019). Intravenous fluid resuscitation is associated with septic endothelial glycocalyx degradation. Crit. Care.

[147] Rehm M., Haller M., Orth V., Kreimeier U., Jacob M., Dressel H., Mayer S., Brechtelsbauser H., Finsterer U. (2001). Changes in blood volume and hematocrit during acute preoperative volume loading with 5% albumin or 6% hetastarch solutions in patients before radical hysterectomy. Anesthesiology.

[148] Nisanevich V., Felsenstein I., Almogy G., Weissman C., Einav S., Matot I. (2005). Effect of intraoperative fluid management on outcome after intraabdominal surgery. Anesthesiology.

[149] Brandstrup B., Tonnesen H., Beier-Holgersen R., Hjortso E., Ording H., Lindorff-Larsen K., Rasmussen M.s., Lanng C., Wallin L., Iversen L.H. (2003). Effects of intravenous fluid restriction on postoperative complications: Comparison of two perioperative fluid regimens: A randomized assessor-blinded multicenter trial. Ann. Surg..

[150] Banerjee S., Mwangi J.G., Stanley T.K., Mitra R., Ebong E.E. (2021). Regeneration and Assessment of the Endothelial Glycocalyx To Address Cardiovascular Disease. Ind. Eng. Chem. Res..

[151] Barelli S., Alberio L. (2018). The role of plasma transfusion in massive bleeding: Protecting the endothelial glycocalyx?. Front. Med..

[152] Wu F., Peng Z., Park P.W., Kozar R.A. (2017). Loss of Syndecan-1 Abrogates the Pulmonary Protective Phenotype Induced by Plasma After Hemorrhagic Shock. Shock.

[153] Khawar H., Kelley W., Stevens J.B., Guzman N. (2021). Fresh Frozen Plasma (FFP). StatPearls.

[154] Caraceni P., Tufoni M., Bonavita M.E. (2013). Clinical use of albumin. Blood Transfus. = Trasfus. Sangue..

[155] Jacob M., Rehm M., Loetsch M., Paul J.O., Bruegger U.W., Conzen P., Becker B.F. (2007). The endothelial glycocalyx prefers albumin for evoking shear stress-induced, nitric oxide-mediated coronary dilatation. J. Vasc. Res..

[156] Jacob M., Bruegger D., Rehm M., Stoeckelhuber M., Welsch U., Conzen P., Becker B.F. (2007). The endothelial glycocalyx affords compatibility of Starling’s principle and high cardiac interstitial albumin levels. Cardiovasc. Res..

[157] Zeng Y., Adamson R.H., Curry F.-R.E., Tarbell J.M. (2014). Sphingosine-1-phosphate protects endothelial glycocalyx by inhibiting syndecan-1 shedding. Am. J. Physiol.-Heart Circul. Physiol..

[158] Zeng Y., Liu X.-H., Tarbell J., Fu B. (2015). Sphingosine 1-phosphateinduced synthesis of glycocalyx on endothelial cells. Exp. Cell Res..

[159] Jacob M., Paul O., Mehringer L., Chappell D., Rehm M., Welsch U., Kaczmarek I., Conzen P., Becker B.F. (2009). Albumin augmentation improves condition of guinea pig hearts after 4 h of cold ischemia. Transplantation.

[160] Adamson R.H., Clark J.F., Radeva M., Kheirolomoom A., Ferrara K.W., Curry F.E. (2014). Albumin modulates S1P delivery from red blood cells in perfused microvessels: Mechanism of the protein effect. Am. J. Physiol. Heart Circul. Physiol..

[161] Maceyka M., Harikumar K.B., Milstien S., Spiegel S. (2012). Sphingosine-1-phosphate signaling and its role in disease. Trends Cell Biol..

[162] Triantafyllidi H., Benas D., Vlachos S., Vlastos D., Pavlidis G., Schoinas A., Varoudi M., Birmpa D., Moutsatsou P., Lekakis J. (2018). HDL cholesterol levels and endothelial glycocalyx integrity in treated hypertensive patients. J. Clin. Hypertens..

[163] Morrish N.J., Wang S.L., Stevens L.K., Fuller J.H., Keen H. (2001). Mortality and causes of death in the WHO Multinational Study of Vascular Disease in Diabetes. Diabetologia.

[164] Kaur G., Harris N.R. (2023). Endothelial glycocalyx in retina, hyperglycemia, and diabetic retinopathy. Am. J. Physiol. Cell Physiol..

[165] Nieuwdorp M., van Haeften T.W., Gouverneur M.C., Mooij H.L., van Lieshout M.H., Levi M., Meijers J.C., Holleman F., Hoekstra J.B., Vink H. (2006). Loss of endothelial glycocalyx during acute hyperglycemia coincides with endothelial dysfunction and coagulation activation in vivo. Diabetes.

[166] Aldecoa C., Llau J.V., Nuvials X., Artigas A. (2020). Role of albumin in the preservation of endothelial glycocalyx integrity and the microcirculation: A review. Ann. Intensive Care.

[167] Zuurbier C.J., Demirci C., Koeman A., Vink H., Ince C. (2005). Short-term hyperglycemia increases endothelial glycocalyx permeability and acutely decreases lineal density of capillaries with flowing red blood cells. J. Appl. Physiol..

[168] O’Hora T.R., Markos F., Wiernsperger N.F., Noble M.I. (2012). Metformin causes nitric oxide-mediated dilatation in a shorter time than insulin in the iliac artery of the anesthetized pig. J. Cardiovasc. Pharmacol..

[169] Cooper S., Teoh H., Campeau M.A., Verma S., Leask R.L. (2019). Empagliflozin restores the integrity of the endothelial glycocalyx in vitro. Mol. Cell Biochem..

[170] Marsh G., Waugh R.E. (2013). Quantifying the mechanical properties of the endothelial glycocalyx with atomic force microscopy. J. Visualized Exp..

[171] Chia P.Y., Teo A., Yeo T.W. (2020). Overview of the Assessment of Endothelial Function in Humans. Front. Med..

[172] Sardu C., Paolisso P., Sacra C., Mauro C., Minicucci F., Portoghese M., Rizzo M.R., Barbieri M., Sasso F.C., D’Onofrio N. (2019). Effects of Metformin Therapy on Coronary Endothelial Dysfunction in Patients with Prediabetes with Stable Angina and Nonobstructive Coronary Artery Stenosis: The CODYCE Multicenter Prospective Study. Diabetes Care.

[173] Nafisa A., Gray S.G., Cao Y., Wang T., Xu S., Wattoo F.H., Barras M., Cohen N., Kamato D., Little P.J. (2018). Endothelial function and dysfunction: Impact of metformin. Pharmacol. Ther..

[174] Inzucchi S.E. (2005). Metformin and heart failure: Innocent until proven guilty. Diabetes Care.

[175] Inzucchi S.E., Masoudi F.A., McGuire D.K. (2007). Metformin in heart failure. Diabetes Care.

[176] Song J.W., Zullo J.A., Liveris D., Dragovich M., Zhang X.F., Goligorsky M.S. (2017). Therapeutic Restoration of Endothelial Glycocalyx in Sepsis. J. Pharmacol. Exp. Ther..

[177] Broekhuizen L.N., Lemkes B.A., Mooij H.L., Meuwese M.C., Verberne H., Holleman F., Schlingemann R.O., Nieuwdorp M., Stroes E.S.G., Vink H. (2010). Effect of sulodexide on endothelial glycocalyx and vascular permeability in patients with type 2 diabetes mellitus. Diabetologia.

[178] Lasierra-Cirujeda J., Coronel P., Aza M.J., Gimeno M. (2010). Use of sulodexide in patients with peripheral vascular disease. J. Blood Med..

[179] Weiss R., Niecestro R., Raz I. (2007). The role of sulodexide in the treatment of diabetic nephropathy. Drugs.

[180] Gambaro G., Kinalska I., Oksa A., Pont’uch P., Hertlova M., Olsovsky J., Manitius J., Fedele D., Czekalski S., Perusicova J. (2002). Oral sulodexide reduces albuminuria in microalbuminuric and macroalbuminuric type 1 and type 2 diabetic patients: The Di.N.A.S. randomized trial. J. Am. Soc. Nephrol..

[181] Drake-Holland A.J., Noble M.I. (2012). Update on the important new drug target in cardiovascular medicine—The vascular glycocalyx. Cardiovasc. Hematol. Disord. Drug. Targets.

[182] Nieuwdorp M., Mooij H.L., Kroon J., Atasever B., Spaan J.A., Ince C., Holleman F., Diamant M., Heine R.J., Hoekstra J.B.L. (2006). Endothelial glycocalyx damage coincides with microalbuminuria in type 1 diabetes. Diabetes.

[183] Salmon A.H.J., Satchell S.C. (2012). Endothelial glycocalyx dysfunction in disease: Albuminuria and increased microvascular permeability. J. Pathol..

[184] Salmon A.H.J., Ferguson J.K., Burford J.L., Gevorgyan H., Nakano D., Harper S.J., Bates D.O., Peti-Peterdi J. (2012). Loss of the endothelial glycocalyx links albuminuria and vascular dysfunction. J. Am. Soc. Nephrol..

[185] Lipowsky H.H., Lescanic A. (2017). Inhibition of inflammation induced shedding of the endothelial glycocalyx with low molecular weight heparin. Microvasc. Res..

[186] VanTeeffelen J.W., Brands J., Stroes E.S., Vink H. (2007). Endothelial glycocalyx: Sweet shield of blood vessels. Trends Cardiovasc. Med..

[187] Hoppensteadt D.A., Fareed J. (2014). Pharmacological profile of sulodexide. Int. Angiol..

[188] He Z., Du X., Wu Y., Hua L., Wan L., Yan N. (2019). Simvastatin promotes endothelial dysfunction by activating the Wnt/betacatenin pathway under oxidative stress. Int. J. Mol. Med..

[189] Meuwese M.C., Mooij H.L., Nieuwdorp M., van Lith B., Marck R., Vink H., Kastelein J.J.P., Stroes E.S.G. (2009). Partial recovery of the endothelial glycocalyx upon rosuvastatin therapy in patients with heterozygous familial hypercholesterolemia. J. Lipid. Res..

[190] Uchimido R., Schmidt E.P., Shapiro N.I. (2019). The glycocalyx: A novel diagnostic and therapeutic target in sepsis. Crit. Care.

[191] Triantafyllou C., Nikolaou M., Ikonomidis I., Bamias G., Kouretas D., Andreadou I., Tsoumani M., Thymis J., Papaconstantinou I. (2021). Effects of Anti-Inflammatory Treatment and Surgical Intervention on Endothelial Glycocalyx, Peripheral and Coronary Microcirculatory Function and Myocardial Deformation in Inflammatory Bowel Disease Patients: A Two-Arms Two-Stage Clinical Trial. Diagnostics.

[192] Steppan J., Hofer S., Funke B., Brenner T., Henrich M., Martin E., Weitz J., Hofmann U., Weigand M.A. (2011). Sepsis and major abdominal surgery lead to flaking of the endothelial glycocalix. J. Surg. Res..

[193] Nelson A., Berkestedt I., Schmidtchen A., Ljunggren L., Bodelsson M. (2008). Increased levels of glycosaminoglycans during septic shock: Relation to mortality and the antibacterial actions of plasma. Shock.

[194] Sallisalmi M., Tenhunen J., Yang R., Oksala N., Pettila V. (2012). Vascular adhesion protein-1 and syndecan-1 in septic shock. Acta. Anaesthesiol. Scand..

[195] Henry C.B., Duling B.R. (2000). TNF-alpha increases entry of macromolecules into luminal endothelial cell glycocalyx. Am. J. Physiol. Heart Circ. Physiol..

[196] Chappell D., Hofmann-Kiefer K., Jacob M., Rehm M., Briegel J., Welsch U., Conzen P., Becker B.F. (2009). TNF-alpha induced shedding of the endothelial glycocalyx is prevented by hydrocortisone and antithrombin. Basic Res. Cardiol..

[197] Nieuwdorp M., Meuwese M.C., Mooij H.L., van Lieshout M.H.P., Hayden A., Levi M., Meijers J.C.M., Ince C., Kastelein J.J.P., Vink H. (2009). Tumor necrosis factor-alpha inhibition protects against endotoxin-induced endothelial glycocalyx perturbation. Atherosclerosis.

[198] Chappell D., Jacob M., Hofmann-Kiefer K., Bruegger D., Rehm M., Conzen P., Welsch U., Becker B.F. (2007). Hydrocortisone preserves the vascular barrier by protecting the endothelial glycocalyx. Anesthesiology.

[199] Kilger E., Weis F., Briegel J., Frey L., Goetz A.E., Reuter D., Nagy A., Schuetz A., Lamm P., Knoll A. (2003). Stress doses of hydrocortisone reduce severe systemic inflammatory response syndrome and improve early outcome in a risk group of patients after cardiac surgery. Crit. Care Med..

[200] Weis F., Kilger E., Roozendaal B., de Quervain D.J.-F., Lamm P., Schmidt M., Schmolz M., Briegel J., Schelling G. (2006). Stress doses of hydrocortisone reduce chronic stress symptoms and improve health-related quality of life in high-risk patients after cardiac surgery: A randomized study. J. Thorac. Cardiovasc. Surg..

[201] Dellinger R.P., Levy M.M., Rhodes A., Annane D., Gerlach H., Opal S.M., Sevransky J.E., Spring C.L., Douglas I.S., Jaeschke R. (2013). Surviving Sepsis Campaign: International guidelines for management of severe sepsis and septic shock, 2012. Intensive Care Med..

[202] Orringer E.P., Casella J.F., Ataga K.I., Koshy M., Adams-Graves P., Luchtman-Jones L., Wun T., Watanabe M., Shafer F., Kutlar A. (2001). Purified Poloxamer 188 for Treatment of Acute Vaso-occlusive Crisis of Sickle Cell DiseaseA Randomized Controlled Trial. JAMA.

[203] Torres Filho I.P., Torres L.N., Salgado C., Dubick M.A. (2017). Novel Adjunct Drugs Reverse Endothelial Glycocalyx Damage After Hemorrhagic Shock in Rats. Shock.

[204] Ikonomidis I., Pavlidis G., Katsimbri P., Lambadiari V., Parissis J., Andreadou I., Tsoumani M., Boumpas D., Kouretas D., Iliodromitis E. (2020). Tocilizumab improves oxidative stress and endothelial glycocalyx: A mechanism that may explain the effects of biological treatment on COVID-19. Food Chem. Toxicol..

[205] Ashry N.A., Abdelaziz R.R., Suddek G.M. (2020). The potential effect of imatinib against hypercholesterolemia induced atherosclerosis, endothelial dysfunction and hepatic injury in rabbits. Life Sci..

[206] Hayakawa M., Kudo D., Saito S., Uchino S., Yamakawa K., Iizuka Y., Sanui M., Takimoto K., Mayumi T., Ono K. (2016). Antithrombin supplementation and mortality in sepsis-induced disseminated intravascular coagulation: A multicenter retrospective observational study. Shock.

[207] Chappell D., Jacob M., Hofmann-Kiefer K., Rehm M., Welsch U., Conzen P., Becker B.F. (2009). Antithrombin reduces shedding of the endothelial glycocalyx following ischaemia/reperfusion. Cardiovasc. Res..

[208] ElSaadani M., Ahmed S.M., Jacovides C., Lopez A., Johnson V.E., Kaplan L.J., Schwab C.W., Smith D.H., Pascual J.L. (2021). Antithrombin III ameliorates post-traumatic brain injury cerebral leukocyte mobilization enhancing recovery of blood brain barrier integrity. J. Trauma. Acute. Care Surg..

[209] Yini S., Heng Z., Xin A., Xiaochun M. (2015). Effect of unfractionated heparin on endothelial glycocalyx in a septic shock model. Acta. Anaesthesiol. Scand..

[210] VanTeeffelen J.W.G.E., Brands J., Jansen C., Spaan J.A.E., Vink H. (2007). Heparin Impairs Glycocalyx Barrier Properties and Attenuates Shear Dependent Vasodilation in Mice. Hypertension.

[211] Karlsson K., Marklund S.L. (1987). Heparin-induced release of extracellular superoxide dismutase to human blood plasma. Biochem. J..

[212] Myrup B., Yokoyama H., Kristiansen O.P., Ostergaard P.B., Olivecrona T. (2004). Release of endothelium-associated proteins into blood by injection of heparin in normal subjects and in patients with Type 1 diabetes. Diabetic. Med..

[213] Annecke T., Chappell D., Chen C., Jacob M., Welsch U., Sommerhoff C.P., Rehm M., Conzen P.F., Becker B.F. (2010). Sevoflurane preserves the endothelial glycocalyx against ischaemia-reperfusion injury. Br. J. Anaesth..

[214] Kim H.J., Kim E., Baek S.-H., Kim H.Y., Kim J.-Y., Park J., Choi E.-J. (2018). Sevoflurane did not show better protective effect on endothelial glycocalyx layer compared to propofol during lung resection surgery with one lung ventilation. J. Thorac. Dis..

[215] Maldonado F., Morales D., Gutierrez R., Barahona M., Cerda O., Caceres M. (2020). Effect of sevoflurane and propofol on tourniquet-induced endothelial damage: A pilot randomized controlled trial for knee-ligament surgery. BMC Anesthesiol..

[216] Kim N.Y., Kim K.J., Lee K.Y., Shin H.J., Cho J., Nam D.J., Kim S.Y. (2021). Effect of volatile and total intravenous anesthesia on syndecan-1 shedding after minimally invasive gastrectomy: A randomized trial. Sci. Rep..

[217] Fang F.Q., Sun J.H., Wu Q.L., Feng L.Y., Fan Y.X., Ye J.X., Gao W., He G.L., Wang W.J. (2021). Protective effect of sevoflurane on vascular endothelial glycocalyx in patients undergoing heart valve surgery: A randomised controlled trial. Eur. J. Anaesthesiol..

[218] Ahlers O., Nachtigall I., Lenze J., Goldmann A., Schulte E., Hohne C., Fritz G., Keh D. (2008). Intraoperative thoracic epidural anaesthesia attenuates stress-induced immunosuppression in patients undergoing major abdominal surgery. Br. J. Anaesth..

[219] Chappell D., Jacob M., Hofmann-Kiefer K., Conzen P., Rehm M. (2008). A rational approach to perioperative fluid management. Anesthesiology.

[220] Holte K., Kehlet H. (2002). Epidural anaesthesia and analgesia—Effects on surgical stress responses and implications for postoperative nutrition. Clin. Nutr..

[221] Annecke T., Rehm M., Bruegger D., Kubitz J.C., Kemming G.I., Stoeckelhuber M., Becker B.F., Conzen P.F. (2012). Ischemia–reperfusion-induced unmeasured anion generation and glycocalyx shedding: Sevoflurane versus propofol anesthesia. J. Investig. Surg..

